# Expulsion of Symbiotic Algae during Feeding by the Green Hydra – a Mechanism for Regulating Symbiont Density?

**DOI:** 10.1371/journal.pone.0002603

**Published:** 2008-07-02

**Authors:** Yelena Fishman, Eliahu Zlotkin, Daniel Sher

**Affiliations:** Department of Cell and Animal Biology, Silberman Institute of Life Sciences, The Hebrew University, Jerusalem, Israel; University of Sheffield, United Kingdom

## Abstract

**Background:**

Algal-cnidarian symbiosis is one of the main factors contributing to the success of cnidarians, and is crucial for the maintenance of coral reefs. While loss of the symbionts (such as in coral bleaching) may cause the death of the cnidarian host, over-proliferation of the algae may also harm the host. Thus, there is a need for the host to regulate the population density of its symbionts. In the green hydra, *Chlorohydra viridissima*, the density of symbiotic algae may be controlled through host modulation of the algal cell cycle. Alternatively, *Chlorohydra* may actively expel their endosymbionts, although this phenomenon has only been observed under experimentally contrived stress conditions.

**Principal Findings:**

We show, using light and electron microscopy, that *Chlorohydra* actively expel endosymbiotic algal cells during predatory feeding on *Artemia*. This expulsion occurs as part of the apocrine mode of secretion from the endodermal digestive cells, but may also occur via an independent exocytotic mechanism.

**Significance:**

Our results demonstrate, for the first time, active expulsion of endosymbiotic algae from cnidarians under natural conditions. We suggest this phenomenon may represent a mechanism whereby cnidarians can expel excess symbiotic algae when an alternative form of nutrition is available in the form of prey.

## Introduction

The endosymbiosis between marine multicellular organisms (most notably cnidarians such as corals and sea anemones) and unicellular symbiotic algae allows for highly productive ecosystems such as coral reefs to thrive in oligotrophic, nutrient-poor oceans [1]. The break-down of this symbiosis can lead to mass mortality of both the host coral and, probably, the symbiotic algae. Such a break-down occurs, for example, during coral bleaching [1], [2]. The long-term stability of such a mutualistic association requires that the needs of both the host and symbiont be maintained. From the point of view of the host cnidarian, one requirement is that the density of the photosynthetic algae found within its endodermal cells needs to be regulated, since over-population of the algae can damage the host, for example through over-accumulation of photosynthetically-derived reactive oxygen species [3], [4].

Three main mechanisms have been proposed whereby the host cnidarian regulates the density of its algal symbionts: regulation of algae growth or division by the host, expulsion of excess algae or their intracellular digestion. Many studies have focused on the role of host-derived factors in the regulation of algal cell cycle [5]–[10]. In contrast, the role of digestion of excess algae is not thought to be important [11], and the expulsion of algae has been directly demonstrated only under stress conditions [11], [12]. Therefore, while the expulsion of algal cells is believed to be important for the regulation of symbiont density [13], how and when this expulsion occurs under natural conditions remains unclear.

## Results and Discussion

**Figure 1:**
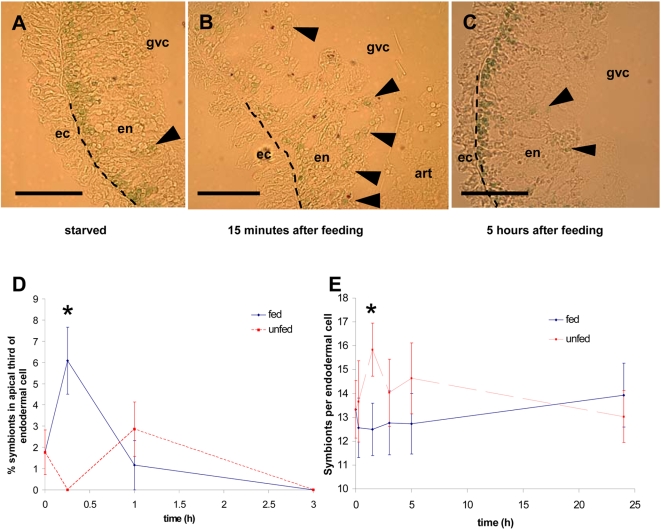
Many symbiotic algae are observed in the apical part of *Chlorohydra viridissima* endodermal digestive cells following feeding with *Artemia*. The symbiotic algae can be seen as small (∼10 µm) green spheres, and those found in the apical part of the cells are marked by arrowheads. A) Unfed *Chlorohydra*; B) *Chlorohydra* 15 minutes after feeding; C) *Chlorohydra* 5 hours after feeding. en = endoderm, ec = ectoderm, gvc = gastrovascular cavity, art = artemia. The dashed line between the endoderm and the mesoderm denotes the mesoglea. Bar = 50 µm. D) A quantitative analysis of the number of symbiotic algae in the apical third of the endoderm, at different times after feeding. Values and error bars represent averages and SE from 4–6 animals. There number of symbionts in the apical part of the cell was weakly affected by time (ANOVA, F = 5.067, p = 0.025) and time by treatment (F = 4.267, p = 0.04) but strongly affected by the treatment (F = 26.667, p<0.001). The difference between the fed and unfed animals is highly significant 15 minutes after feeding (Student's two-tailed t-test, p = 0.0048, represented by * in the figure), and is not significant at other time points. E) Reduction in the number of algal symbionts per endodermal cell in fed compared to unfed animals, 15 minutes after feeding. Values and error bars represent averages and SE from 30 endodermal cells per time point. The number of symbionts was not affected by time (ANOVA, F = 0.438, p = 0.781), and the effect of treatment was marginally significant (F = 3.76, p = 0.053). There was a significant difference between fed and unfed animals after 15 minutes (Student's two tailed t-test, t = 2.686, p = 0.009, represented by * in the figure) but this difference was not significant at other time points.

In a previous study [14], we have demonstrated using immunohistochemistry that the green hydra, *Chlorohydra viridissima*, secretes pore forming proteins from the endodermal digestive cells into the gastrovascular cavity (GVC) during prey digestion, using an apocrine mechanism of secretion. This mechanism involves budding off of part of the apical end of the cell into the GVC, followed by lysis of the budded “aposome” and release of its content [15]. In the sections used for immunohistochemistry, we noticed that the location of the symbiotic algae within the endodermal digestive cells changed during the various stages of feeding. In unfed animals (previously starved for 72 hours), most of the symbiotic algae were found at the base of the endodermal digestive cells (Figures 1A, 1D), as reported by numerous other studies [11], [16]. However, within fifteen minutes of prey capture and ingestion by the green hydra, many of the algae were observed outside the basal region of the cells, in the middle or apical parts (Figures 1B, 1D). At later time points (one, three, five and eight hours after feeding) most of the algae were again observed in the basal region of the cells (Figures 1C, 1D and data not shown). Additionally, free algae could occasionally be seen within the GVC of the *Chlorohydra* following ingestion of the prey *Artemia*. This shift in location of the symbiotic algae, from the base of the endoderm towards the apical part, was consistently seen in three independent experiments. Thus, during feeding, some of the algae appear to migrate towards the apical part of the endodermal cells, close to the GVC, and are probably secreted into the GVC itself. In support of the latter hypothesis, namely that the symbionts at the apical part of the cell are secreted into the GVC, we have observed a small but statistically significant reduction in the number of symbionts in isolated endodermal cells from fed animals compared to control, unfed animals, 15 minutes after feeding (Figure 1E). It is noteworthy that McAuley observed a similar apical migration of algae during experimental bleaching of Green Hydra [11], and that Bossert and Dunn [6] observed a small reduction in the number of algae per host cell in the first few hours after feeding.

**Figure 2:**
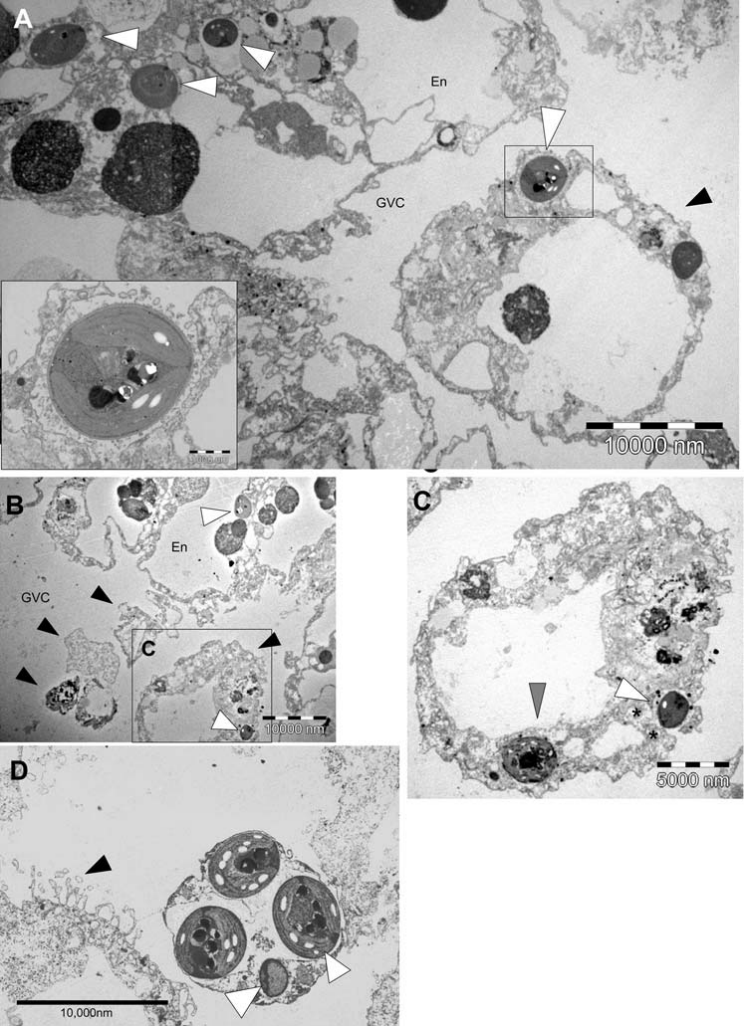
Expulsion of symbiotic algae by apocrine secretion and exocytosis during feeding by *Chlorohydra*. en = endoderm, gvc = gastrovascular cavity. A) A general view of the apical part of *Chlorohydra* endoderm, 15 minutes after feeding. A large membrane-bound “aposome” is seen within the GVC, adjacent to the apical membrane of an endodermal digestive cell. (black arrowhead). A symbiotic alga is in the process of exocytosis from the aposome (enlarged in the inset), and several others are seen in the apical part of the endoderm (white arrowheads). Bar = 10 µm (1 µm in the inset). B) Expulsion of alga during apocrine secretion, 15 minutes after feeding. Note heterogenous aposomes within the GVC (black arrowheads), one of which contains an alga (algae are marked by white arrowheads). Bar = 10 µm. C) Enlargement of the aposome marked by an square in B. The aposome contains one intact alga (in the process of exocytosis, white arrowhead), as well as possibly another being digested (grey arrowhead). * = mitochondria. Bar = 5 µm. D) An aposome containing four symbiotic algae within the GVC (white arrowheads). Note the microvilli seen on the apical membrane of an endodermal cell (black arrowhead). Bar = 10 µm.

To further characterize this phenomenon, we used transmission electron microscopy (TEM) to study the ultrastructure of the algae-containing *Chlorohydra* cells before and during feeding. In agreement with the observations described above, TEM revealed that symbiotic algae could often be seen in the apical part of the hydra endodermal digestive cells, fifteen minutes after ingestion of prey (Figure 2A). Algae were rarely observed in the apical part of the endoderm in sections of starved *Chlorohydra*. Large, membrane bound fragments could be seen within the GVC of fed *Chlorohydra*, adjacent to the apical part of the endoderm. We have previously shown that similar cellular debris contain Hydralysins, pore forming proteins synthesized by the endodermal digestive cells, and probably represent “aposomes” – fragments of the endodermal digestive cells pinched off during apocrine secretion [14], [15]. As shown in Figure 2, several of these aposomes contained symbiotic algae, supporting our previous hypothesis that these cellular debris indeed originate from the endodermal digestive cells. Additionally, these data reveal that the symbiotic algae can be expelled by *Chlorohydra* through pinching off of large parts of the digestive cells during feeding. It is noteworthy that a morphologically similar phenomenon (expulsion of symbionts in large cellular debris) has been observed by Gates and co-workers in sea anemones and corals in response to heat and cold stress, although in this case it was suggested that a holocrine mechanism is involved, whereby an entire cell ruptures, releasing its contents [12]. The inclusion of algae in aposomes pinched off during feeding is not necessarily a specific mechanism for algae expulsion, but rather may be a by-product of a natural event occurring during feeding and digestion in *Chlorohydra* and possibly other cnidarians. If this is indeed the case, the “decision” to expel a specific alga using this mechanism is probably taken when the alga begins to migrate from the base of the endodermal digestive cell towards the apex and GVC.

In addition to the apocrine mechanism of algae expulsion described above, we also observed active exocytosis of symbiotic algae from the apical part of the endodermal digestive cells, and even from aposomes already separated from the main part of the cell body (Figure 2). The exocytosis was characterized by a fusion of the host-derived membrane surrounding the symbiont (“symbiosome”) with the plasma membrane bordering the GVC (inset in Figure 2A). In contrast to the expulsion of the symbionts by inclusion in aposomes, the specific exocytosis of the algae implies a specific recognition of the alga as an object to be expelled from the cnidarian cell.

The present observations show, for the first time, that a symbiotic cnidarian actively expels some of its symbiotic algae during predatory feeding. This is in agreement with the work of Bossert and Dunn, who observed a small reduction in the number of algae per hydra cell four hours after feeding (Figure 1 in [6]). The time point at which we observed the expulsion of symbiotic algae by *Chlorohydra*, during or immediately after feeding, may have several important advantages. Firstly, it may allow the green hydra to make “an informed choice”, replacing one source of energy (photosynthetic products transferred from the symbiotic algae) with another (prey) based on the immediate availability of caught prey. Secondly, it is known that both the algae and the host cells undergo mitosis several hours after feeding [5], [7], [9]. Expelling the algae before they undergo mitosis may mean that fewer algae need to be actively expelled, costing the green hydra less metabolic energy. Finally, the algae are expelled into the GVC while the hydra is in the middle of actively digesting prey, with the GVC already full of the various digestive enzymes and compounds. Thus, the expelled algae may also be digested, providing additional nutrients to the host without additional metabolic cost. It remains to be seen whether other cnidarians also expel symbiotic algae during feeding, and how this mechanism is used together with others (such as regulation of algal cell cycle by the cnidarian) to maintain an optimal symbiont density.

## Materials and Methods

### Animals

*Chlorohydra viridissima* were originally obtained from the Volcani Center for Agricultural Research, Israel. They were maintained in glass dishes in M medium [17] at 20°C with a 12∶12 light∶dark cycle. The *Chlorohydra* were fed three times a week with freshly hatched nauplii of *Artemia salina*. Unless otherwise mentioned, prior to experimentation the animals were starved for three days.

### Light microscopy

*Chlorohydra* were fed 2–3 day old nauplii of *Artemia salina*, transferred carefully to an eppendorf tube and fixed in 4% paraformaldehyde. They were washed extensively M-medium, transferred to PBST (Phosphate Buffered Saline containing 0.1% Tween-20), infiltrated with 30% sucrose in PBST, immersed in Tissue-Tek medium and frozen on dry ice or liquid nitrogen. 7 µm sections were taken using a Leica CM1850 cryostat (Wetzlar, Germany), and mounted in glycerol. Micrographs were takes with a Zeiss Axioskop-2 microscope (Carl Zeiss, Jena, Germany) equipped with an Olympus DP10 CCD camera (Tokyo, Japan).

To quantify the location of the symbiontic algae within the endoderm, we measured the distance of algae from the mesoglea and divided this by the width of the endoderm at that region. We performed this analysis for algae along 100 µm of the mesoglea, and repeated this analysis on sections from 4–6 animals per experimental time point.

To count the number of symbionts per endodermal cell, *Chlorohydra* were macerated at various times after feeding according to David [18], and the number of symbionts counted in 30 endodermal cells per experimental time point. As the results were not normally distributed (Kolmogrov–Smirnov) the data were square-root transformed prior to their anaylsis. Statistical analysis was performed using SPSS ver 12.

### Transmission Electron Microscopy

*Chlorohydra* (either unfed or 15 minutes after feeding) were fixed with 4% paraformaldehyde +1% glutaraldehyde in M-medium, pH 7.4 at 4°C overnight, followed by postfixation for one hour with 1% w/v OsO4. The *Chlorohydra* were dehydrated through an ethanol series, followed by 100% propyleneoxide, embedding in Agar-100 resin (Agar Scientific) and polymerization for 3 days at 60°C. 700Å sections were obtained using an Ultrotome-3 (LKB-Prodikter). Thin sections were stained with saturated aqueous uranyl acetate and lead citrate solutions and observed with Tecnai 12 (Phillips) Trasnmission Electron microscope equipped with MegaView II CCD camera and AnalySIS® version 3.0 software (SoftImaging System GmbH) or with a JEOL CX-100 Transmission Electron Microscope.
